# Editorial: Women in onconephrology: 2022

**DOI:** 10.3389/fneph.2023.1240195

**Published:** 2023-08-01

**Authors:** Ala Abudayyeh, Sabine Karam

**Affiliations:** ^1^ Section of Nephrology, The University of Texas MD Anderson Cancer Center, Houston, TX, United States; ^2^ Division of Nephrology and Hypertension, University of Minnesota, Minneapolis, MN, United States

**Keywords:** onconephrology, women, cancer, immune check inhibitor, acute kidney injury

With the advancement in cancer therapy, there has been a dramatic improvement in the survival of cancer patients with nonetheless increased complexity regarding their care. Cancer therapeutics continue to be innovative by evolving from the use of traditional chemotherapies to that of targeted therapies based on gene mutations in the tumor and immunotherapies where we harness the immune system against tumors; all of which are associated with potentially significant renal toxicities. In addition, with the high dependence on adequate kidney function to be eligible for newer available therapies and clinical trials, it has been realized that many aspects of nephrology, including electrolytes abnormalities, acute kidney injury (AKI), and chronic kidney disease (CKD) among others, occurring in an oncology patient represent important aspects of a cancer patient’s care. With the appreciation of the intersecting paths of both cancer medicine and nephrology, the term “onconephrology” was coined. Several initiatives followed the birth of this specialized term such as an American Society of Nephrology (ASN) forum in 2011 under the leadership of Dr. Abdulla Salahudeen (1, 2), dedicated journal issues, onconephrology symposiums locally and internationally, an ASN onconephrology pre-course as part of Kidney Week and numerous onconephrology curriculums. Today, a dedicated fellowship with at least four programs in the United States and one program in Canada offers the opportunity to train in the field. Moreover, there is a journal solely dedicated to the specialty, and a Kidney Disease: Improving Global Outcomes (KDIGO) Controversies Conference on onconephrology recently occurred (3). Furthermore, with this immense recognition of onconephrology as a specialty and rising numbers of onconephrologists, the American Society of Onconephrology was formed in 2021 under the leadership of Drs. Shruti Gupta and Kenar Jhaveri. It is nonetheless worth mentioning that long before this formal recognition, kidney complications within and from cancer were already a matter of great concern among the scientific community, with concerning reports published since the 1950s as the first case of contrast-associated AKI in a patient with myelomatosis was reported in 1954 (4) and the recognition of the associated nephrotoxicity of cisplatin (5). This Research Topic of “*Women in Onconephrology*” aims to highlight the great contributions that prominent female nephrologists have made to the specialty nationally and internationally, pushing the field further and branching new interests such as transplant onconephrology (6–16), with the first example being Thelma B. Dunn, the first woman elected as president of the American Association for Cancer Research. Many of her scientific contributions could be filed today under “onconephrology” (17, 18). In this Research Topic, four articles written by women pioneers investigate forefront topics related to cancer and nephrology, and, most notably, immune checkpoint inhibitor (ICI) uses and associated renal toxicity.

In a review by Miao et al., the authors present an overview of the incidence and risk factors for ICI nephrotoxicity, pathophysiological mechanisms, pathologies, and therapeutic options. They also discuss the controversies surrounding the re-challenge of a patient affected with AKI with ICI and the use of novel biomarkers for the non-invasive diagnosis of ICI-induced kidney injury. They notably point out that while a single biomarker might neither be sensitive nor specific for the diagnosis of acute interstitial nephritis (AIN) induced by ICI, the use of multi-positive biomarkers could certainly be more sensitive and guide the decision of performing a kidney biopsy, a modality that Fenoglio et al. advocate for use in their article where they review the findings of kidney biopsies done in a cohort of 30 patients to diagnose toxicities associated with targeted therapies and ICI. The authors demonstrate that histological findings were useful to initiate a more effective and targeted treatment rather than to empirically default to steroids and/or hold their cancer treatments in the presence of AKI. Furthermore, in this Research Topic, the sensitive topic of using ICI in kidney transplant recipients is thoroughly approached. This population has an increased risk of developing cancers, especially non-melanoma skin cancers. The use of ICI to treat kidney transplant patients with skin cancers has been a challenge with an increased risk of rejection which has been noted to be as high as 42% at a median time of 24 days post-ICI (19). Meerhaeghe et al. present a promising case series of 7 kidney transplant patients with advanced cutaneous squamous cell carcinoma (cSCC) in Belgium treated with cemiplimab (a human monoclonal IgG4 antibody against anti-PD-1). The study concluded that the overall response rate was 42.8% and only that one patient had biopsy-proven acute renal allograft rejection with nonetheless complete tumor response. Finally, this Research Topic presents a case report by Qureshi et al. in which the authors present a patient with advanced non-small cell lung cancer and pre-existing antineutrophil cytoplasmic (anti-PR3) antibodies who later develops multi-organ vasculitis after ICI exposure, successfully treated with rituximab, with continued cancer remission for 3 years. The article evaluates the rare incidence of autoimmune induction in the kidney after ICI exposure and the use of rituximab which has been proven to be effective in other published cases.

In conclusion, this Research Topic, “*Women in Onconephrology*”, comprising articles by vibrant women researchers, presents interesting new perspectives in the field most notably related to a better understanding of the use of ICI in patients with cancer and kidney disease but also highlights the numerous gaps that remain to be filled. It also illustrates the role of immunological triggers for the onset of glomerular diseases through the example of immunotherapy. Although not addressed in this Research Topic, it would be also important to address in the future the differences between women and men in terms of risk, disease presentation, and treatment effects in “onconephrology”, as these are important features in “*Women in Onconephrology*”.

## Author contributions

All authors listed have made a substantial, direct, and intellectual contribution to the work and approved it for publication.
